# Characterization of *Vibrio fluvialis qnrVC5* Gene in Native and Heterologous Hosts: Synergy of *qnrVC5* with other Determinants in Conferring Quinolone Resistance

**DOI:** 10.3389/fmicb.2016.00146

**Published:** 2016-02-15

**Authors:** Kittappa Vinothkumar, G. N. Kumar, Ashima K. Bhardwaj

**Affiliations:** ^1^Molecular Biology of Diseases, Department of Human Health and Diseases, School of Biological Sciences and Biotechnology, Indian Institute of Advanced ResearchGandhinagar, India; ^2^Department of Bio-Chemistry, Faculty of Science, The Maharaja Sayajirao University of BarodaVadodara, India

**Keywords:** gene cassettes, plasmids, superintegron, quinolone susceptibility

## Abstract

Resistance of various pathogens toward quinolones has emerged as a serious threat to combat infections. Analysis of plethora of genes and resistance mechanisms associated with quinolone resistance reveals chromosome-borne and transferable determinants. *qnr* genes have been found to be responsible for transferable quinolone resistance. In the present work, a new allele *qnrVC5* earlier reported in *Vibrio fluvialis* from this laboratory was characterized in detail for its sequence, genetic context and propensity to decrease the susceptibility for quinolones. The study has revealed persistence of *qnrVC5* in clinical isolates of *V. fluvialis* from Kolkata region through the years 2002–2006. *qnrVC5* existed in the form of a gene cassette with the open reading frame being flanked by an upstream promoter and a downstream *V. cholerae* repeat region suggestive of its superintegron origin. Sequence analysis of different *qnrVC* alleles showed that *qnrVC5* was closely related to *qnrVC2* and *qnrVC4* and these alleles were associated with *V. cholerae* repeats. In contrast, *qnrVC1, qnrVC3*, and *qnrVC6* belonging to another group were associated with *V. parahaemolyticus* repeats. The gene manifested its activity in native *V. fluvialis* host as well as in *Escherichia coli* transformants harboring it by elevating the MIC toward various quinolones by twofold to eightfold. In combination with other quinolone resistance factors such as topoisomerase mutations and *aac(6’)-Ib-cr* gene, *qnrVC5* gene product contributed toward higher quinolone resistance displayed by *V. fluvialis* isolates. Silencing of the gene using antisense peptide nucleic acid sensitized the *V. fluvialis* parent isolates toward ciprofloxacin. Recombinant QnrVC5 vividly demonstrated its role in conferring quinolone resistance. *qnrVC5* gene, its synergistic effect and global dissemination should be perceived as a menace for quinolone-based therapies.

## Introduction

*Vibrio fluvialis* is known to cause severe cholera-like diarrhea in humans and has been perceived as an emerging pathogen (Bhattacharjee et al., 2010; Ramamurthy et al., 2014). Diarrheal illnesses caused by this kind of bacteria are generally treated using quinolone class of antibiotics. Increase in the reports of emergence of multi drug resistant (MDR) *V. fluvialis*, showing considerable resistance to quinolones has been a public health concern (Srinivasan et al., 2006; Singh et al., 2012; Ramamurthy et al., 2014). Reduced susceptibility to quinolones in bacteria is mediated by factors such as mutations in the genes for DNA gyrase and topoisomerase IV (the drug targets), eﬄux activity, Qnr proteins and the inactivation of drug by quinolone-modifying enzyme [AAC (6’) Ib-Cr; Bhardwaj et al., 2014; Kim and Hooper, 2014]. The above mentioned mechanisms may work alone or in combination. The synergistic action of these mechanisms helps the pathogen to achieve higher-level of resistance toward quinolones (Baranwal et al., 2002; Srinivasan et al., 2006; Rushdy et al., 2013; Zhu et al., 2013).

Qnr proteins are pentapeptide repeat proteins (PRPs) which protect DNA gyrase from quinolone action (Tran and Jacoby, 2002). *qnr*, a plasmid-mediated horizontally transferable gene conferring quinolone resistance, was first discovered in a plasmid from *Klebsiella pneumoniae* in 1998 (Martinez-Martinez et al., 1998). Thereon, different variants of *qnr* genes such as *qnrA, qnrB, qnrC, qnrD*, and *qnrS* were reported in different pathogens from different parts of the world (Strahilevitz et al., 2009). Chromosomal *qnr*-like genes such as *qnrVC*, Pp*qnr*, Vp*qnr*, and Sm*qnr* were also reported from *V. cholerae* (Fonseca et al., 2008), *Photobacterium profundum* (Poirel et al., 2005a), *V. parahaemolyticus* (Saga et al., 2005), and *Stenotrophomonas maltophilia* (Sanchez et al., 2008), respectively. Qnr proteins cause low-level resistance to quinolones, facilitating the emergence of resistant mutants. In combination with other mechanisms of quinolone resistance such as topoisomerase mutations and eﬄux action, Qnr proteins can help the pathogens to achieve clinical breakpoints of quinolone resistance (Martinez-Martinez et al., 2003; Jeong et al., 2008).

Till date, structures for PRPs such as MfpA, EfsQnr, QnrB1, and AhQnr have been solved (Hegde et al., 2005, 2011; Vetting et al., 2011; Xiong et al., 2011). QnrB1 (from *K. pneumoniae*) and AhQnr (from *Aeromonas hydrophila)* possessed a right handed quadrilateral β-helix, which is typical of PRPs and encompassed coils with two loops (loop A and loop B) extending outward from the regular structure, interrupting the β-helix turn. Unlike the PRP structures of MfpA and EfsQnr from Gram-positive bacteria *Mycobacterium tuberculosis* and *Enterococcus faecalis*, respectively, QnrB1 and AhQnr possessed these two loops. It was established that these two loops played a vital role in the interaction of Qnr and DNA gyrase subunits (GyrA and GyrB; Xiong et al., 2011). Loop A was found to interact with GyrA “tower” whereas loop B was found to interact with GyrB TOPRIM (Topoisomerase-Primase) domains. This also indicated that the mechanisms of interaction of MfpA and EfsQnr with DNA gyrase were different from that of QnrB1 and AhQnr. These loops are found to be conserved among all plasmid-based Qnr variants and some chromosome-borne Qnr proteins (Vetting et al., 2011).

A former belief based on the mode of action of MfpA and EfsQnr explains that Qnr protein binds with DNA gyrase and prevents the formation of cleaved complex. Qnr protein does not interact with the quinolones and therefore renders resistance to drugs indirectly (Hegde et al., 2005, 2011). As the structure of MfpA and EfsQnr varied from QnrB1 and AhQnr, their mode of action should also principally vary. The model proposed by Vetting et al. suggested that QnrB1 protein binds to and destabilizes the topoisomerase-quinolone-DNA cleavage complex, which eventually results in the release of quinolone and religation of DNA. After this process, the Qnr protein would be released and the active form of topoisomerase would be regenerated (Vetting et al., 2011).

Emergence of transferable *qnrVC* alleles in *Vibrionaceae* family and other bacterial species aggravated the hysteria on quinolone resistance (Fonseca and Vicente, 2013; Pons et al., 2013). So far, seven *qnrVC* alleles (named as *qnrVC1* to *qnrVC7*) have been reported from different parts of the globe^1^. These alleles are found as gene cassettes, equipped with all the elements necessary for their mobility, incorporation and expression such as *attC* sites and their own cassette-specific promoter (Fonseca and Vicente, 2013).

Previous studies from this laboratory have revealed the presence of a *qnrVC*-like gene in the plasmids of three *V. fluvialis* clinical isolates, BD146, L10734, and L9978 (Rajpara et al., 2009; Singh et al., 2012) and this allele was named *qnrVC5* by Fonseca and Vicente in 2013 (Fonseca and Vicente, 2013). The plasmid that harbored *qnrVC5* in *V. fluvialis* BD146 also carried the gene encoding trimethoprim resistance (*dfrVI*) and showed 99% identity with pVN84 from *V. cholerae* O1 isolated from Vietnam (Rajpara et al., 2009) and plasmid from *V. parahaemolyticus* V110 isolated from Hong Kong, China (Liu and Chen, 2013; Bhardwaj, 2015). The plasmid from *V. parahaemolyticus* V110 possessed *qnrVC5* allele whereas plasmid pVN84 carried *qnrVC2*, a non-functional form of *qnrVC5* due to the presence of several internal stop codons (Fonseca et al., 2008; Fonseca and Vicente, 2013). From the above discussion and other reports, it is amply clear that *qnrVC* genes are disseminated globally and are likely to play a vital role in quinolone resistance due to their wide dispersal (Rajpara et al., 2009; Kim et al., 2010; Kumar and Thomas, 2011; Fonseca and Vicente, 2013; Liu and Chen, 2013; Pons et al., 2013; Bhardwaj, 2015). Since *qnrVC5* allele was first reported from this laboratory, it was of prime interest to decipher its role in conferring resistance to quinolones. Therefore, the present study was undertaken to understand the features of this gene/its product *in silico*. Another focus of the study was to functionally characterize QnrVC5 protein in native *V. fluvialis* host and heterologous *E. coli* host. Results reflected the role of *qnrVC5* in conferring resistance to quinolones and its synergy with other quinolone resistance factors such as mutations in topoisomerase genes and *aac(6’) Ib-cr* gene.

## Materials and Methods

### Bacterial Strains and Plasmids

Clinical isolates of *V. fluvialis* BD146, L13828, L10734, L9978, and L15318 were obtained from patients suffering from acute cholera-like diarrhea admitted to The Infectious Diseases Hospital, Kolkata during 2002–2006 (Kind gift from Dr. T. Ramamurthy, National Institute of Cholera and Enteric Diseases, Kolkata), and have been used in earlier studies (Rajpara et al., 2009; Singh et al., 2012). Plasmid pET 28a (Novagen) was used for expression of *qnrVC5* gene. *E. coli* JM109 was used for transformation experiments and *E. coli* BL21 (λDE3) was used for recombinant protein expression studies.

### *In Silico* Analysis

The DNA sequence analyses were done using BLAST tool available at NCBI site^2^ and phylogenetic tree was constructed using maximum-likelihood method in MEGA6 (Tamura et al., 2013). Softberry-BPROM, a promoter prediction tool was used to find the promoters and other regulatory elements in *qnrVC5* gene cassette ^3^. The structure of QnrVC5 was predicted by I-TASSER server using automated mode, as it employs hierarchical method for protein structure and function prediction using multiple threading approach based on structural templates from PDB (Zhang, 2008; Roy et al., 2010, 2012).

### Peptide Nucleic Acid (PNA)

The anti-*qnrVC* peptide-PNA [H-(KFF)_3_K-O-ccattttctagccct-NH_2_] complementary to the region encompassing ribosomal binding site and start codon of *qnrVC5* gene was designed to silence the *qnrVC5* gene at RNA level. The PNA was conjugated with cell penetrating peptide (KFF)_3_ to enhance the permeability of this antisense oligonucleotide across the cell membrane of bacteria (Good et al., 2001). The peptide-PNA was synthesized by PANAGENE (Daejeon, South Korea) and the lyophilized PNA oligomer was dissolved in sterile water as per manufacturer’s instructions.

### DNA Preparations

The preparations of genomic and plasmid DNA of *V. fluvialis* were done as described earlier (Thungapathra et al., 2002). Plasmid Mini kit or Maxi kit (Qiagen) was used for plasmid DNA isolation according to the manufacturer’s instructions.

### Polymerase Chain Reaction (PCR) and Reverse Transcription PCR (RT-PCR)

Genomic DNA (200 ng) or plasmid DNA (10–50 ng) were used as templates for PCR reactions. PCR reactions were performed using the protocol described earlier (Singh et al., 2012). Primers used for PCR experiments were qnrVC-F 5′-CGC**GGATCC**ATGGATAAAACAGACCAG-3′ and qnrVC-R 5′-CCG**CTCGAG**TTAGTCAGGAACTACTAT-3′. These primers incorporated sites (in bold) for *Bam*HI and *Xho*I restriction enzymes that were used for cloning in the expression vector pET28a. Primers used to amplify the entire *qnrVC5* gene cassette, encompassing the upstream and downstream sequences along with coding region, were qnrVCcas-F 5′-CGTATAGAAAGCGTTATGTG-3′ and qnrVCcas-R 5′-CTGCTGCCATAATGGATAT-3′. Each PCR consisted of an initial denaturation at 94°C for 5 min, followed by 30 amplification cycles, each involving an initial denaturation at 94°C for 0.5 min followed by annealing and extension steps. Annealing condition for qnrVC-F and qnrVC-R primer pair was 65°C for 0.5 min, and for qnrVCcas-F and qnrVCcas-R primer pair was 60°C for 0.5 min. For both the above reactions, extension was performed at 72°C for 1 min and final polymerization was carried out at 72°C for 10 min. Taq polymerase (Fermentas) was used and reactions were performed in T100 thermal cycler (Bio-Rad Laboratories). Purification of PCR products was performed using QIA-quick PCR purification kit (Qiagen) as per the manufacturer’s instructions.

RT-PCR was carried out to confirm the expression of *qnrVC5* gene and *aac(6’) Ib-cr* gene in the native isolates of *V. fluvialis* and their *E. coli* JM109 transformants. Total RNA was isolated from *V. fluvialis* isolates and their *E. coli* transformants, using RNeasy bacteria mini kit (Qiagen) as per the manufacturer’s instructions. The protocol consisted of growing the cultures in LB medium followed by treatment with lysozyme and proteinase K for cell lysis and RNA was purified from the lysate using RNeasy mini spin columns. The RNA preparations were subsequently treated with DNaseI (Fermentas) to remove the genomic DNA contamination. RT-PCR was carried out using Qiagen one step RT-PCR kit following manufacturer’s instructions. Each RT-PCR reaction mixture consisted of 10 μl of 5X Qiagen 1-step RT-PCR buffer, 2.0 μl of dNTP mix containing 2.5 mM of each dNTP, 50.0 pmol of each primer, 2.0 μl of Qiagen 1-step RT-PCR enzyme mix and RNase free water to a final volume of 49.0 μl. The 1.0 μl template RNA (0.1 μg μl^-1^) was added to make the final reaction volume of 50.0 μl. The primer pair qnrVC-F and qnrVC-R, mentioned above, were used for RT-PCR of *qnrVC5* transcripts, whereas AG-F 5′- TGACCAACTGCAACGATTCC -3′ and AG-R 5′- ACCCATAGAGCATCGCAAGGT -3′ were used for RT-PCR of *aac(6’)Ib-cr* transcripts. Each RT-PCR experiment consisted of reverse transcription step at 50°C for 30 min and an initial denaturation at 95°C for 15 min, followed by 30 amplification cycles, each consisting of a denaturation step at 94°C for 0.5 min followed by annealing and extension steps. The annealing condition for *qnrVC5* amplification was 60°C for 0.5 min and for *aac(6’) Ib-cr* amplification was 64°C for 0.5 min. Extension was performed at 72°C for 1 min and final polymerization was carried out at 72°C for 10 min. The reactions were performed in T100 thermal cycler (Bio-Rad Laboratories).

### Gene Cloning and Protein Expression

Vector pET28a and the amplicon of *qnrVC5* (from *V. fluvialis* BD146) each were separately double-digested with *Bam*HI and *Xho*I. Subsequent to digestion, the dephosphorylated vector and *qnrVC5* insert were ligated, the ligation mixture was electroporated in *E. coli* JM109 cells and the transformed cells were plated on LB plates containing kanamycin (50 μg ml^-1^) to obtain the recombinants. Expression of QnrVC5 protein was studied by induction of *E. coli* BL21 (λDE3) cells harboring the recombinant clones with 1mM IPTG for 2 h at 37°C, followed by SDS-PAGE analysis of total cell lysates. The protein band corresponding to QnrVC5 was excised from the gel and subjected to trypsin digestion. The authenticity of this protein was then confirmed by peptide mass fingerprinting on Bruker Ultraflex III MALDI instrument. The protein identification was done through Mascot software.

### Minimum Inhibitory Concentration (MIC) Assays

Twofold dilution method was used to determine the MIC of various quinolones for native *V. fluvialis* clinical isolates, *E. coli* transformants and QnrVC5 recombinant as described previously with minor modifications described below (Mohanty et al., 2012).

#### (i) MIC assay for native *V. fluvialis* clinical isolates and their *E. coli* JM109 transformants

Overnight grown colonies of *V. fluvialis* were inoculated in 5 ml LB and grown at 37°C till the optical density at 600 nm reached 0.1. This culture was used as inoculum for MIC assay. Concentration of the test drug was diluted twofold in Muller Hinton Broth (MHB). Fifty microliters of inoculum was added to 2 ml of each drug concentration in a 24-well cell culture plate and incubated at 37°C for 18 h. MIC was read as the lowest concentration of the drug where no growth was observed. The assays were repeated at least three times. The MIC of *E. coli* JM109 transformants (obtained from the plasmid preparation derived from the native *V. fluvialis* host) was determined by the same method except that the transformants were selected in LB medium containing ampicillin (25 μg ml^-1^) before inoculating into the MIC assay plate.

#### (ii) MIC assay for QnrVC5 recombinants

The recombinant plasmid was transformed into *E. coli* BL21 (λDE3) cells and the transformants were selected on LB agar containing kanamycin (50 μg ml^-1^). *E. coli* BL21 (λDE3) cells carrying vector pET28a was used as a control. Both the recombinant and the control were induced with 1mM IPTG for 2 h. Subsequently, the induced cultures were adjusted to the optical density of 0.1 at 600 nm and used as inoculum for MIC assays. The concentration of the test drug was diluted twofold in MHB containing kanamycin (50 μg ml^-1^) and IPTG (1 mM). Fifty microliters of inoculum was added to 2 ml of each drug concentration in a 24-well cell culture plate and incubated at 37°C for 18 h to determine the MIC value as described above. The assays were repeated at least three times.

#### (iii) MIC assay for *V. fluvialis* isolates in presence of PNA

MIC of ciprofloxacin was determined for *V. fluvialis* isolates (BD146, L10734, L9978, and L15318) using the same twofold dilution method described above, but in a 96-well polypropylene plate in a total assay volume of 100 μl with 5 μl of cultures as inoculum. The effect of PNA in reducing the MIC of ciprofloxacin was tested as described previously (Jeon and Zhang, 2009). The assay was initially done with *V. fluvialis* BD146 to check the effect on MIC with increasing concentrations of PNA (0, 2, 4, and 6 μM). Subsequent to that, assays were performed without and with PNA (4 μM) for all the above mentioned *V. fluvialis* isolates.

### Mutant Prevention Concentration (MPC) Assay

Mutant prevention concentration assay was performed as described previously with minor modifications (Marcusson et al., 2005). Hundred microliters of overnight grown culture of each of the *V. fluvialis* isolates (BD146, L15318, L10734, L9978, and L13828) was inoculated in 25 ml LB and grown at 37°C until the optical density at 600 nm reached 1.0 (∼10^9^ cells/ml). The culture was centrifuged at 8000 rpm for 5 min and the pellet was resuspended in LB to contain ≥ 10^10^ cells/ml. Subsequently, 200 μl of resuspended culture was spread on the Muller Hinton Agar (MHA) plates containing defined concentrations of ciprofloxacin. Each strain was tested with six concentrations of ciprofloxacin starting from their MIC (i.e., 1X, 2X, 4X, 8X, 16X, 32X MIC). The plates were incubated at 37°C for 96 h and MPC was recorded as the lowest concentration of ciprofloxacin that prevented the emergence of mutant colonies. The assays were individually performed three times.

## Results

### Presence of *qnrVC5* Gene in *V. fluvialis* Isolates, Characterization of the Gene, and its Flanking Genetic Environment

The plasmid pBD146 (GenBank accession no. EU574928) obtained from a clinical isolate of *V. fluvialis* BD146, 2002, was earlier reported to harbor a *qnr* gene (GenBank accession no. JN408080; Rajpara et al., 2009). In another report, the same gene was also found in plasmid preparations from two *V. fluvialis* isolates L10734 (GenBank accession no. JN571550) and L9978 (GenBank accession no. JN571549; Singh et al., 2012) and termed as *qnrVC*-like gene. Later, it was named *qnrVC5* allele (Fonseca and Vicente, 2013). The sequence encoded a protein of 218 amino acids with two domains of 11 and 32 pentapeptide repeats bridged by a glycine residue.

**FIGURE 1:**
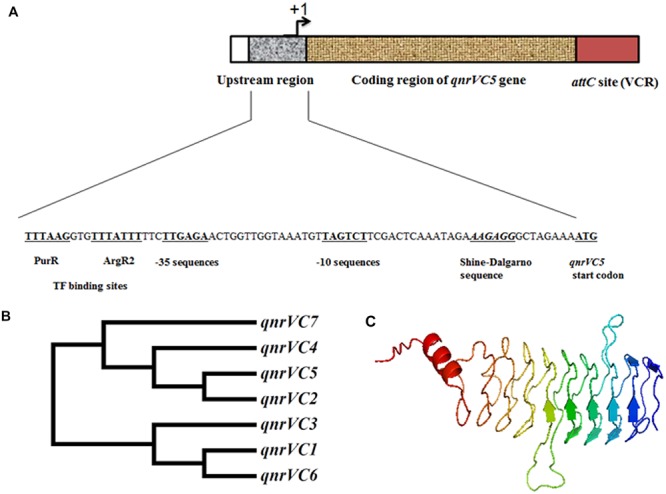
**The genetic environment, homology and structure prediction of the gene/gene product of *qnrVC5* gene from *V. fluvialis* isolate BD146. (A)** The structure of *qnrVC5* gene cassette in pBD146 with canonical promoter sequence and VCR recombination site, at the upstream and downstream positions of the coding region, respectively. **(B)** Dendrogram of *qnrVC5* allele with other *qnrVC* alleles, showing the close relationship of *qnrVC5* with *qnrVC2* and *qnrVC4.* **(C)** Structure prediction of QnrVC5 using I-TASSER server, showing right handed quadrilateral β-helix forming 10 coils, interrupted by two loops.

Based on the sequence of pBD146, it was evident that *qnrVC5* gene in *V. fluvialis* isolate BD146 was present in the form of a gene cassette where ORF was flanked with a downstream recombination site corresponding to *V. cholerae* repeat (VCR) region and an upstream internal promoter (P_qnrV C_) with canonical sequence (**Figure 1A**). Most interestingly, upstream of –10 and –35 sequences of the P_qnrV C_ promoter, binding sites for PurR (purine metabolism repressor) and ArgR2 (arginine metabolism regulator) were also predicted in this cassette using Softberry – BPROM online tool (**Figure 1A**). To further characterize the gene sequence from other two isolates (L10734 and L9978), primers qnrVCcas-F and qnrVCcas-R were designed based on the sequence flanking *qnrVC5* in pBD146. PCR carried out using these primers revealed that the same length of gene cassette was also amplified from these two isolates suggestive of the similar gene organization in these three bacterial isolates.

### Sequence Analysis, Homology, and Structure Prediction for *qnrVC5* Gene/Protein

At nucleotide level, the ORF of *qnrVC5* allele shared 99% identity with *qnrVC4* and *qnrVC2*, and 97% identity with *qnrVC7*. *qnrVC5* sequence was found to have 76% identity with *qnrVC1* and 75% with both *qnrVC3* and *qnrVC6* alleles. The phylogenetic tree for all these *qnrVC* genes showed that *qnrVC1, qnrVC3*, and *qnrVC6* belonged to the same group, whereas *qnrVC2, qnrVC4, qnrVC5*, and *qnrVC7* formed another group (**Figure 1B**). This clearly showed that *qnrVC5* was closely related to *qnrVC2* and *qnrVC4*, which corroborated the earlier findings (Fonseca and Vicente, 2013). In addition to this, it was observed that among all the *qnrVC* alleles listed out by Fonseca and Vicente, (Fonseca and Vicente, 2013) *qnrVC1, qnrVC3*, and *qnrVC6* alleles were associated with *V. parahaemolyticus* repeats (VPRs) whereas *qnrVC2, qnrVC4*, and *qnrVC5* alleles were associated with VCRs, indicating different lineage for these two groups of *qnrVC* alleles. Three dimensional structure prediction of QnrVC5 protein using I-TASSER server depicted the structure of a typical PRP, threading into β helical folds interrupted by two loops (**Figure 1C**). QnrVC5 protein encompassed 10 coils with two loops (loop A and loop B) extending outward from the regular structure, interrupting the β-helix turn. Loop A and loop B from QnrVC5 shared 25 and 50% sequence homology, respectively, with the corresponding loops of QnrB1. Structure predicted from the threading experiments on I-TASSER server was based on the templates of O-methyl transferase (PDB ID: 3DUL) from *Bacillus cereus* and QnrB1 (PDB ID: 2XTY and 2XTW) from *K. pneumoniae*. The structural analogs of the predicted structure were QnrB1 (PDB ID: 2XTW), AhQnr (PDB ID: 3PSS) from *A. hydrophila* and *O*-methyl transferase (PDB ID: 3DUL).

### Contribution of *qnrVC5* Gene in Quinolone Resistance of their Native *V. fluvialis* Host

The effect of *qnrVC5* gene in elevating the MIC and MPC of quinolones in *V. fluvialis* isolates was determined. All the *V. fluvialis* isolates harboring *qnrVC5* (BD146, L10734, and L9978) were subjected to MIC assays with nalidixic acid, norfloxacin, ciprofloxacin, and ofloxacin and MPC assays with ciprofloxacin. Among these three strains, L9978 possessed *qnrVC5* as the only detected quinolone resistance determinant whereas rest two were having other factors along with *qnrVC5* gene (**Table 1**). *V. fluvialis* BD146 had GyrA S83I, ParC S85L mutations and *aac (6’)-Ib-cr* gene as the quinolone resistance factors in addition to *qnrVC5* gene. *V. fluvialis* L10734 had GyrA S83I and ParC S85L mutations along with *qnrVC5* gene (**Table 1**). The quinolone resistant *V. fluvialis* strain L15318 (having GyrA S83I and ParC S85L mutations and lacking *qnrVC5)* and quinolone sensitive *V. fluvialis* strain L13828 were included as controls (**Table 1**). The MIC and MPC values of *V. fluvialis* isolates for quinolones have been mentioned in **Table 1**. It was observed that when compared to the sensitive strain L13828, the *qnrVC5*-bearing strain L9978 showed about twofold to fourfold elevation in MIC of tested quinolones and twofold elevation in MPC of ciprofloxacin. When compared to L15318, BD146 showed twofold to fourfold increase in MIC of ciprofloxacin and slight increase (<twofold) in MIC of nalidixic acid, norfloxacin, and ofloxacin presumably due to additional presence of PMQR determinants *qnrVC5* gene and *aac (6’)-Ib-cr* gene apart from GyrA and parC mutations. This result also indicated that *qnrVC5* and *aac (6’)-Ib-cr* determinants may chiefly contribute toward resistance to ciprofloxacin. As expected, the MPC of ciprofloxacin was also elevated eightfold in *V. fluvialis* BD146 when compared to L15318 and L10734. Though it may be difficult to compare MIC/MPC of non-isogenic strains, synergy between different resistance factors was evident in majority of cases except L10734 and L15318. Similarly, the lower MIC obtained for nalidixic acid in L10734 as compared to L15318 could not be explained.

**Table 1:** Quinolone susceptibility of *V. fluvialis* strains and their corresponding *E. coli* transformants.

Strain/transformant	Quinolone resistance determinants	MIC (μg ml^-1^)				MPC of Ciprofloxacin (μg ml^-1^)
		Nalidixic acid	Norfloxacin	Ciprofloxacin	Ofloxacin	
***V. fluvialis***						
BD146	GyrA S83I, ParC S85L, *qnrVC5* and *aac(6’)Ib-cr*	1500	17.5–20	10	10	320
L15318	GyrA S83I, ParC S85L	1000	15	2.5–5	8	40
L10734	GyrA S83I, ParC S85L and *qnrVC5*	125	10	2.5	8	40
L9978	*qnrVC5*	2	1.25	0.312	0.5	1.25–5
L13828	None	0.75	0.312	0.156	0.312	0.625–2.5
***E. coli* JM109**						
BD146 transformant (7.5 kb+)	*qnrVC5* and *aac(6’)Ib-cr*	400	4	0.5	1	ND
BD146 transformant (7.5 kb–)	*aac(6’)Ib-cr*	200	2	0.25	0.5	ND
L10734 transformant	*qnrVC5*	200	2	0.25	1	ND
L9978 transformant	*qnrVC5*	400	4	0.5	2	ND
JM109 (non-transformant)	None	50	1	0.125	0.5	ND

*ND, Not done.*

### Contribution of *qnrVC5* Gene in Quinolone Resistance of *E. coli* Transformants

In the previous studies from our laboratory, plasmid preparations from *V. fluvialis* isolates BD146 (Rajpara et al., 2009), L10734, and L9978 (Singh et al., 2012) were transformed into *E. coli* JM109 to elucidate their transferable traits. *E. coli* transformants of these three *V. fluvialis* plasmid preparations harboring *qnrVC5* gene were utilized in this study to find the effect of this gene in elevating the MIC of quinolones. For BD146, two types of *E. coli* transformants were observed on the basis of presence or absence of a 7.5 kb plasmid pBD146 harboring *qnrVC5* gene. The transformants that possessed 7.5 kb plasmid as well as another low copy number plasmid [bearing *aac(6’) Ib-cr* gene] were termed as 7.5 kb+. On the other hand, the transformants harboring only low copy number plasmid but devoid of the 7.5 kb plasmid were termed as 7.5 kb- (Rajpara et al., 2009). In other words, the transformants 7.5 kb+ possessed both *qnrVC5* and *aac(6’) Ib-cr* genes whereas 7.5 kb- transformants were positive for *aac (6’) Ib-cr* gene only (**Table 1**). Therefore, in the present study, one 7.5 kb+ transformant was selected to study the effect of *qnrVC5* gene in combination with *aac (6’) Ib-cr* and one 7.5 kb- transformant was selected to study the effect of *aac (6’) Ib-cr* alone in quinolone resistance. For similar reasons, transformants of L10734 and L9978 bearing *qnrVC5* gene (Singh et al., 2012) were included to study the effect of *qnrVC5* gene alone. Untransformed *E. coli* JM109 was used as a negative control for the MIC assays. The MIC values for all the four transformants and *E. coli* JM109 are mentioned in **Table 1**. The transformants having *qnrVC5* alone showed twofold to fourfold elevation in MIC of norfloxacin, ciprofloxacin and ofloxacin and fourfold to eightfold increase in the MIC of nalidixic acid when compared to *E. coli* JM109. The 7.5 kb+ transformant having *qnrVC5* along with *aac (6’) Ib-cr* gene showed eightfold increase in MIC of nalidixic acid and fourfold for norfloxacin and ciprofloxacin. The 7.5 kb+ transformant showed twofold increase in the MIC of ofloxacin. The 7.5 kb- transformant having *aac (6’) Ib-cr* gene alone, showed fourfold increase in MIC for nalidixic acid and twofold for norfloxacin and ciprofloxacin but did not show any effect against ofloxacin.

### Expression of *qnrVC5* Gene in Native *V. fluvialis* Host and *E. coli* Transformants

**FIGURE 2:**
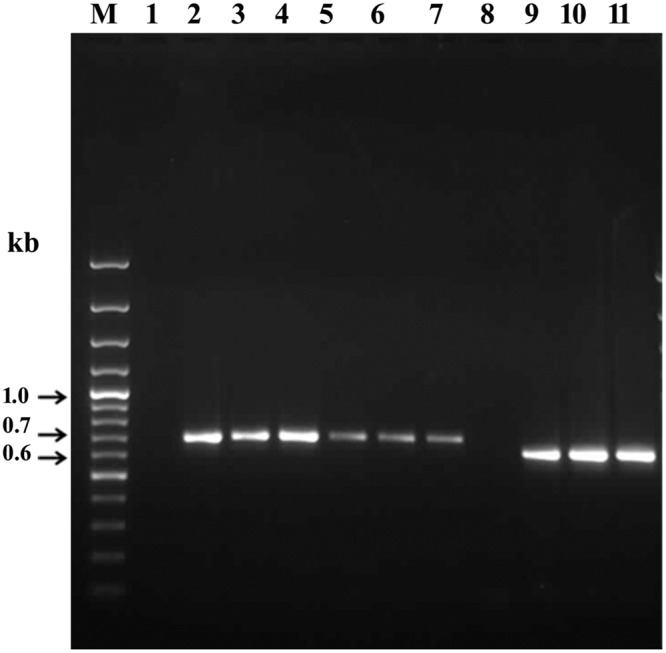
**Expression of *qnrVC5* and *aac(6’) Ib-cr* genes in the native *V. fluvialis* host and in *E. coli* JM109 transformants derived from the plasmids of native hosts.** 100 bp Gene ruler (Fermentas) was used as the marker (M). Lane 1: Negative control (without reverse transcription step) for RT-PCR product of *qnrVC5* transcript (657 bp) from *V. fluvialis* hosts BD146; Lanes 2 to 4: RT-PCR products of *qnrVC5* transcript (657 bp) from *V. fluvialis* hosts BD146, L10734 and L9978, respectively; Lanes 5 to 7: RT-PCR products of *qnrVC5* transcript (657 bp) from transformants of BD146 (7.5 kb+), L10734 and L9978; Lane 8: Negative control (without reverse transcription step) for RT-PCR product of *aac(6’) Ib-cr* transcript (657 bp) from *V. fluvialis* hosts BD146; Lanes 9 to 11: RT-PCR products of *aac(6’) Ib-cr* transcript (608 bp) from *V. fluvialis* BD146 and its transformants (7.5 kb+ and 7.5 kb–), respectively.

RT-PCR was carried out to confirm the expression of *qnrVC5* gene and *aac(6’) Ib-cr* gene in the native host and their *E. coli* transformants (**Figure 2**). In both the cases, genes were expressed with the expected band size of 657 bp for *qnrVC5* (**Figure 2**, Lanes 2 to 7) and 608 bp for *aac(6’) Ib-cr* (**Figure 2**, Lanes 9 to 11). The negative controls without reverse transcription step that were included to ensure the absence of DNA contamination in RNA templates, did not show any band corresponding to the expressed genes. Two such negative controls with BD146 RNA templates for RT-PCR of *qnrVC5* and *aac(6’) Ib-cr* transcripts have been shown (**Figure 2**, Lanes 1 and 8, respectively).

### Effect of PNA Against *qnrVC5* Gene in Native *V. fluvialis* Host

Once the presence of *qnrVC5* RNA was confirmed in the native host, a peptide-PNA against *qnrVC5* RNA was used to silence its expression. MIC of ciprofloxacin for *V. fluvialis* BD146 was determined in the presence of increasing concentrations of PNA (0, 2, 4, and 6 μM). The results showed a steady decrease of MIC values with increasing concentration of PNA with 4 μM of PNA appearing as an effective dose for inhibition. Subsequently, MIC of ciprofloxacin for all the three *qnrVC5-*bearing *V. fluvialis* isolates (BD146, L10734, and L9978) and one *qnrVC5*-lacking *V. fluvialis* isolate L15318 was tested at a final concentration of 4 μM of PNA. Twofold to eightfold decrease in MIC of ciprofloxacin in all the three isolates was observed in the presence of PNA when compared to the control with no PNA (**Table 2**). In *qnrVC5*-lacking *V. fluvialis* isolate L15318, the MIC of ciprofloxacin was similar irrespective of the presence of PNA indicating that PNA did not have any non-specific inhibitory activity (**Table 2**).

**Table 2:** Silencing effect of *qnrVC5* gene in *V. fluvialis* strains.

*V. fluvialis*	MIC of Ciprofloxacin (μg ml^-1^)		Fold in reduction of MIC
	PNA-	PNA+	
BD146	10–20	2.5–5	2–8
L10734	2.5	1.25	2
L9978	0.156	0.078	2
L15318	5	5	0

### Gene Cloning and Recombinant Protein Expression

The *qnrVC5* gene was cloned in pET28a expression vector and the authenticity of the recombinant clones was confirmed. A protein band of ∼27 kDa was found to be overexpressed by the recombinants on 1 mM IPTG induction. This protein band was excised from the SDS-PAGE gel and subjected to trypsin digestion. The peptide mass fingerprinting analysis confirmed it to be QnrVC5.

### Elevation of MIC for quinolones in *qnrVC5* Recombinants

Minimum inhibitory concentration assays were carried out to study the functionality of recombinant QnrVC5 protein. MIC was tested with different generations of quinolones with nalidixic acid representing first generation, norfloxacin, ciprofloxacin, ofloxacin, and levofloxacin representing second generation, sparfloxacin for third generation and moxifloxacin as a representative of fourth generation. Interestingly, recombinant *E. coli* BL21(λDE3) cells were found to show eightfold to more than 64-fold increase in MIC of different quinolones when compared to control having pET28a alone (**Table 3**). QnrVC5 conferred higher resistance toward ciprofloxacin and sparfloxacin as the MICs of these two drugs were elevated to more than 64-fold. MIC of levofloxacin and moxifloxacin were elevated to 64-fold and 32-fold, respectively. QnrVC5 elevated the MIC of both norfloxacin and ofloxacin to 16-fold and nalidixic acid to eightfold (**Table 3**).

**Table 3:** MIC of quinolones for pET-qnrVC5 clone.

Quinolones	MIC (μg ml^-1^)		Fold in elevation of MIC
	*E. coli* (BL21 λDE3) (pET-qnrVC5 clone)	*E. coli* (BL21 λDE3) (pET28a)	
Nalidixic acid	5	0.625	8
Norfloxacin	0.125	0.0078	16
Ciprofloxacin	0.0156	<0.000243	>64
Ofloxacin	0.0625	0.0039	16
Levofloxacin	0.0312	0.000487	64
Sparfloxacin	0.125	<0.00195	>64
Moxifloxacin	0.125	0.0039	32

## Discussion

In the studies aimed at unraveling the molecular mechanisms of drug resistance in the clinical isolates of *V. fluvialis*, a new allele named *qnrVC5* was reported from this laboratory (Rajpara et al., 2009; Singh et al., 2012). The current study was intended to characterize this plasmid-associated gene from those clinical isolates, for its role in conferring protection/resistance toward quinolones. *qnrVC5* ORF was found as a gene cassette with its own promoter with canonical sequence and *attC* site which was identical to *V. cholerae* repeats (VCR). BLAST search indicated that this gene cassette showed 99% identity with *qnrVC2* gene cassette found in the plasmid pVN84 harbored by *V. cholerae* O1, isolated from Vietnam during 2004 as well as *qnrVC5* gene cassette found in another plasmid present in *V. parahaemolyticus* V110, isolated from Hong Kong during 2010. The *qnrVC5* gene cassette also showed 99% identity to *qnrVC4* gene cassettes carried in class I integrons of *Aeromonas caviae, A. hydrophila, E. coli*, and *Salmonella enterica* and 98% identity to *qnrVC4* gene in class I integron of *A. punctata*. This indicated evolutionary relationship among *qnrVC2, qnrVC5*, and *qnrVC4* gene cassettes and their dissemination in various species of bacteria through mobile genetic elements. *qnrVC5* gene cassette showed 97% homology with a *qnr* cassette found in super integron (SI) on the small chromosome of *V. cholerae* MS6 (Okada et al., 2014). The VCR region downstream of *qnrVC5* ORF, showed identity to the stretch of sequences present in the chromosome of various *Vibrio* and *Shewanella* species. This indicated the possible exchange of various resistance conferring genes or gene cassettes among these bacterial species of *Shewanellaceae* and *Vibrionaceae* families in their environmental vicinity. This observation supported the hypothesis by Poirel et al. (2005a,b) that these two families could be the reservoirs of *qnr* genes. This additionally supported the chromosomal origin of *qnr* alleles. The presence of regulatory motif for DNA-binding proteins PurR and ArgR indicated the possible involvement of these elements in controlling the expression of *qnrVC5* gene. This was suggestive of the probable biological function of Qnr proteins in relation to purine and arginine metabolism or indicate that *qnrVC5* might be one of the potentially co-regulated set of genes interlinked with amino acid and nucleotide metabolism.

The antimicrobial susceptibility assays clearly showed the involvement of *qnrVC5* gene in conferring resistance to different quinolones. Compared to the sensitive *V. fluvialis* strain (L13828), *qnrVC5-*bearing-isolates resisted the quinolone action by virtue of *qnrVC5* alone as in L9978 or in unison with other mechanisms as in BD146 and L10734. The *qnrVC5-* bearing- plasmids from the parent strain, transformed to the heterologous host *E. coli* JM109, proved the role played by the gene in reducing susceptibility toward quinolones. Hence, the *qnrVC5* gene could effectively express its traits in different bacterial hosts as these plasmids disseminated through horizontal gene transfer. Silencing of the gene increased the susceptibility of the parent strains toward ciprofloxacin from twofold to eightfold, again confirming the role of *qnrVC5* in drug resistance.

The results of MIC and MPC assays were vivid representations of synergy between various quinolone resistance determinants (with some exceptions) with BD146 as a carrier of all the three determinants and rest of the isolates as carriers of either one or two determinants. These results also clearly indicated the major role of topoisomerase mutations in susceptibility for quinolones with *qnrVC5* or *aac (6’)Ib-cr*, playing the role of an apprentice. Though the level of resistance conferred by *qnrVC5* for ciprofloxacin was low, it apparently helped the pathogen in elevating the MIC and MPC of ciprofloxacin for *V. fluvialis* strains (BD146 and L9978) thus extending the mutant selection window for ciprofloxacin in these strains. This is likely to help in the selection of more mutants at higher concentrations of drug, by enhancing the survival ability of the pathogen (Strahilevitz et al., 2009). However, as described in the results section, there were anomalies in this synergistic design and therefore could not be explained. Perhaps, comparisons of susceptibility trends in non-isogenic strains may not be very straight-forward and may reflect a complex interplay of many known and yet unknown genetic factors.

Recently, the role of *qnrVC1* gene located in a chromosomal integron in clinical and environmental *Pseudomonas aeruginosa* isolates, in conferring resistance to quinolones, has been described (Belotti et al., 2015). *qnrVC1* gene with its promoter was cloned in *E. coli* and *P. aeruginosa* and shown to confer variable resistance to quinolones. In the present study, T7 promoter-based overexpression of *qnrVC5* gene was utilized to study the gene function in isolation which was not possible in native *V. fluvialis* isolates. MIC study with the recombinant reflected that the potency of this gene in conferring resistance is significant and variable resistance was observed for different generations of quinolones.

Using I-TASSER server, structure of QnrVC5 protein was predicted and was found to be similar to that of QnrB1 (Vetting et al., 2011) and AhQnr structure (Xiong et al., 2011) implying that the function of QnrVC5 could also be similar to the above two proteins (i.e., imparting quinolone resistance). The important feature in the structures of QnrB1 and AhQnr is the presence of two loops and their contribution in protection of DNA gyrase from quinolones (Vetting et al., 2011; Xiong et al., 2011). Therefore, the working model of Qnr proteins (PRPs with loops) in quinolone resistance was proposed, in which the Qnr proteins disturb the quinolone-topoisomerase-DNA complex (Vetting et al., 2011; Xiong et al., 2011). Our functional analysis of recombinant QnrVC5 protein also strengthened the above mentioned model. QnrVC5 protein was shown to elevate the MIC of different quinolones by varying degrees. The possible reason for varied elevation in MIC would be the consequence of varying binding orientations of the different quinolones with the topoisomerase-DNA complex. For example, the interactions of nalidixic acid and ciprofloxacin with gyrase differ in drug binding orientation (perhaps reflected in differences in their potency) such that Qnr destabilizes the ciprofloxacin interaction to a greater extent than the nalidixic acid interaction.

## Conclusion

These findings prove that the carriage of *qnrVC* alleles on various mobile genetic elements such as plasmids, integrons, and SXT elements in a variety of organisms of different genera have been disseminating quinolone resistance widely throughout the globe (Fonseca et al., 2008; Rajpara et al., 2009; Kim et al., 2010; Kumar and Thomas, 2011; Fonseca and Vicente, 2013). With the indiscriminate use of quinolones, this can turn out to be both a reason as well as a consequence of the serious problem of multi-drug resistance.

## Authors Contributions

Conceived and designed the experiments: AB and KV. Performed the experiments: KV. Analyzed the data: AB, GK, and KV. Contributed reagents/materials/analysis tools: AB. Wrote the paper: AB, GK, and KV.

## Conflict of Interest Statement

The authors declare that theresearch was conducted in the absence of any commercial or financial relationships that could be construed as a potential conflict of interest.

## Footnotes

1 http://www.lahey.org/qnrstudies/

2 http://blast.ncbi.nlm.nih.gov/Blast.cgi

3 http://linux1.softberry.com/berry.phtml?topic=bprom&group=programs&subgroup=gfindb
